# Construction of machine learning tools to predict threatened miscarriage in the first trimester based on AEA, progesterone and β-hCG in China: a multicentre, observational, case-control study

**DOI:** 10.1186/s12884-022-05025-y

**Published:** 2022-09-09

**Authors:** Jingying Huang, Ping Lv, Yunzhi Lian, Meihua Zhang, Xin Ge, Shuheng Li, Yingxia Pan, Jiangman Zhao, Yue Xu, Hui Tang, Nan Li, Zhishan Zhang

**Affiliations:** 1 Department of Gynaecology, Quanzhou First Hospital Affiliated to Fujian Medical University, Quanzhou, 362000 Fujian China; 2 Department of Obstetrics, Jincheng People’s Hospital, Jincheng, 048000 Shanxi China; 3 Department of Clinical Laboratory, Jincheng People’s Hospital, Jincheng, 048000 Shanxi China; 4 Department of Medical Image, Tengzhou Central People’s Hospital Affiliated to Jining Medical University, Tengzhou, 277500 Shandong China; 5 Department of Pharmacy, Tengzhou Central People’s Hospital Affiliated to Jining Medical University, Tengzhou, 277500 Shandong China; 6 Department of Thyroid &Mammary, Tengzhou Central People’s Hospital Affiliated to Jining Medical University, Tengzhou, 277500 Shandong China; 7 Shanghai Biotecan Pharmaceuticals Co., Ltd, No. 180 Zhangheng Road, Shanghai, 201204 China; 8 Shanghai Biotecan Medical Diagnostics Co., Ltd, Shanghai, 201204 China; 9 Department of Gynaecology, Tengzhou Central People’s Hospital Affiliated to Jining Medical University, Tengzhou, 277500 Shandong China; 10 Department of Clinical Laboratory, Quanzhou First Hospital Affiliated to Fujian Medical University, Quanzhou, 362000 Fujian China

**Keywords:** Endocannabinoid anandamide, Progesterone, β-Human chorionic gonadotrophin, Threatened miscarriage

## Abstract

**Background:**

Endocannabinoid anandamide (AEA), progesterone (P4) and β-human chorionic gonadotrophin (β-hCG) are associated with the threatened miscarriage in the early stage. However, no study has investigated whether combing these three hormones could predict threatened miscarriage. Thus, we aim to establish machine learning models utilizing these three hormones to predict threatened miscarriage risk.

**Methods:**

This is a multicentre, observational, case-control study involving 215 pregnant women. We recruited 119 normal pregnant women and 96 threatened miscarriage pregnant women including 58 women with ongoing pregnancy and 38 women with inevitable miscarriage. P4 and β-hCG levels were detected by chemiluminescence immunoassay assay. The level of AEA was tested by ultra-high-performance liquid chromatography-tandem mass spectrometry. Six predictive machine learning models were established and evaluated by the confusion matrix, area under the receiver operating characteristic (ROC) curve (AUC), accuracy and precision.

**Results:**

The median concentration of AEA was significantly lower in the healthy pregnant women group than that in the threatened miscarriage group, while the median concentration of P4 was significantly higher in the normal pregnancy group than that in the threatened miscarriage group. Only the median level of P4 was significantly lower in the inevitable miscarriage group than that in the ongoing pregnancy group. Moreover, AEA is strongly positively correlated with threatened miscarriage, while P4 is negatively correlated with both threatened miscarriage and inevitable miscarriage. Interestingly, AEA and P4 are negatively correlated with each other. Among six models, logistic regression (LR), support vector machine (SVM) and multilayer perceptron (MLP) models obtained the AUC values of 0.75, 0.70 and 0.70, respectively; and their accuracy and precision were all above 0.60. Among these three models, the LR model showed the highest accuracy (0.65) and precision (0.70) to predict threatened miscarriage.

**Conclusions:**

The LR model showed the highest overall predictive power, thus machine learning combined with the level of AEA, P4 and β-hCG might be a new approach to predict the threatened miscarriage risk in the near feature.

**Supplementary Information:**

The online version contains supplementary material available at 10.1186/s12884-022-05025-y.

## Background

Miscarriage is a common complication in early pregnancy, occurring in around 15% of clinically recognized pregnancies, and approximately 11% women will experience threatened miscarriage [1]. Threatened miscarriage is diagnosed by vaginal bleeding with or without abdominal pain. Surprisingly, 50% pregnancies with threatened miscarriage had an inevitable miscarriage [2].

Progesterone (P4) is secreted by the corpus luteum during pregnancy, which is essential at various stages of pregnancy. The deficiency of P4 in early pregnancy is associated with an increased miscarriage risk [3]. Therefore, P4 supplementation has been used as a treatment for threatened miscarriage to prevent spontaneous pregnancy loss [4].

Human chorionic gonadotrophin (β-hCG) is a glycoprotein secreted by the syncytiotrophoblast. Recent studies demonstrated that the concentration of serum β-hCG in early pregnancy can predict pregnancy outcome [5]. The serum β-hCG increased rapidly in the early stage of pregnancy and showed a linear increase in peak approximately 8 to 10 weeks of the pregnancy, and declined rapidly a few weeks before delivery [6].

Recent animal studies suggest that endocannabinoid anandamide (AEA) is pivotal for both blastocyst development and endometrium implantation, and low AEA levels enhances implantation success [7]. AEA is synthesized by N-acyl phosphatidylethanolamine phospholipase D (NAPE-PLD) and binds both cannabinoid receptors (CB1 and CB2) [8]. AEA can be regulated by the enzyme named fatty acid amide hydrolase (FAAH), which metabolizes AEA into arachidonic acid (AA) and ethanolamine [9]. Studies indicated that women with spontaneous or threatened miscarriage are associated with high AEA levels and low FAAH expression [10, 11]. The plasma AEA levels in women with threatened miscarriage are higher in those who subsequently spontaneously miscarried than in those who had live births [12]. Therefore, it is necessary to develop a reliable early warning method for threatened miscarriage that could lead to early intervention and treatment for threatened miscarriage.

In this study, we recruited 119 normal pregnancy women in their first trimester and 96 women with threatened miscarriage including 58 cases with ongoing pregnancy and 38 cases with inevitable miscarriage. We aim to use machine learning tools combining the level of AEA, P4 and β-hCG to predict the risk of threatened miscarriage.

## Materials and methods

### Patients and study design

This is a multicentre, observational, case-control study. A total of 96 pregnant women with threatened miscarriages were consecutively enrolled according to the inclusion and exclusion criteria from Quanzhou First Hospital, Tengzhou Central People’s Hospital and Jincheng People’s Hospital from August 2017 to May 2019. Meanwhile, 119 normal pregnancy women were randomly selected at the same time, who were matched with threatened miscarriage patients on the basis of age and gestational age in a roughly 1: 1-1.5 case-control ratio.

The inclusion criteria were as follows [13]: i) single intrauterine pregnancy < 13 weeks of gestational age (the diagnose of intrauterine pregnancy was based on clinical assessment and evaluation by ultrasonography); ii) Threatened miscarriage group: women with pregnancy-related vagina bleeding; Normal pregnancy group: women with none pregnancy-related vagina bleeding; iii) age > 20 years. The exclusion criteria were as follows [13, 14]: i) Women with multiple gestations; ii) Women with previous episodes of vagina bleeding or those treated with progesterone for previous vagina bleeding in the current pregnancy; iii) Women diagnosed with missed miscarriage, blighted ovum or planned termination of pregnancy; iv) Women had severe medical disease, such as severe coronary heart disease, stroke or malignant disease; v) Women who lost follow-up.

Informed consent was obtained from each patient participated in the study and the study protocol conforms to the ethical guidelines of the latest version of Declaration of Helsinki. The study protocol has been approved by Ethical Committee of the Quanzhou First Hospital, Tengzhou Central People’s Hospital and Jincheng People’s Hospital.

### Detection of serum β-hCG and P4 levels

The concentrations of serum β-hCG (mIU/mL) and P4 (ng/mL) were tested in the Clinical Pathology Laboratory of the Quanzhou First Hospital, Tengzhou Central People’s Hospital and Jincheng People’s Hospital according to the standard protocols.

### Detection of AEA level

Plasma AEA was extracted and performed as previously described [15]. Briefly, 4 mL blood was collected in EDTA tube and placed on ice. After centrifugation at 1200 g/30 min at 22 °C, 2 mL of plasma was transferred to a glass Kimble scintillation vial (Fisher Scientific, Loughborough, UK) and added 2.5 pmol of deuterium-labelled AEA (AEA-d8; Cayman Chemicals, Ann Arbor, MI, USA). Plasma proteins were mixed with an equal volume of ice-cold acetone followed by centrifugation at 1200 g/10 min at 22 °C. Then the supernatant was transferred to a clean Kimble vial and used in the subsequent steps according to the instructions. The reconstituted mixture was performed by the ultrahigh performance liquid chromatography-tandem mass spectrometry (UPLC-MS/MS) as described [16].

### Predictive models construction

Six machine learning tools were established to predict the threatened miscarriage, including logistic regression (LR) model, random forest (RF) model, extreme gradient boosting (XGboost) model, k-nearest neighbors classifier (KNN) model, multilayer perceptron (MLP) neural network model and support vector machine (SVM) model and combined AEA, P4 and β-hCG by Python (v.3.7.0). All models were chosen default parameters. The patients were randomly allocated into training set. In the training set, k-fold cross-validation (k = 5) was used. K-fold is a common cross validation approach as described [17]. For each model, the evaluation indicators used were the confusion matrix, area under the receiver operating characteristic (ROC) curve (AUC), accuracy and precision.

### Statistical analysis

Continuous variables are presented as the median with interquartile range (*IQR)* because of the non-Gaussian distributions of our data [18]. Continuous variables between the two groups were compared using a nonparametric Mann–Whitney test by GraphPad Prism 8.0 (GraphPad Software, La Jolla, CA, USA). Six machine learning models (KNN, LR, SVM, RF, MLP and XGboost) were performed and evaluated by Python (v.3.7.0). The diagnostic values of the 6 models were assessed by ROC analysis. Correlations among threatened miscarriage, inevitable miscarriage, AEA, P4 and β-hCG were analyzed by Pearson correlation analysis using the “psych” package [19] of *R* studio [20] in *R* software [21]. A *P* < 0*.*05 was considered statistically significant.

## Results

### Comparison of AEA, P4, β-hCG and clinical data between women with healthy pregnancies and threatened miscarriages

A total of 215 pregnant women were recruited, including 119 healthy pregnant women (normal pregnancy group) and 96 pregnant women with threatened miscarriages (threatened miscarriage group). The median concentration with IQR of AEA was significantly lower in the normal pregnancy group than that in the threatened miscarriage group, which is 0.62 (0.30-1.21) nM vs. 1.21 (0.72-1.83) nM. Meanwhile, the median concentration with IQR of P4 was significantly higher in the normal pregnancy group than that in the threatened miscarriage group, which is 21.92 (17.48-27.83) ng/mL vs. 19.53 (13.28-24.21) ng/mL. However, there were no noticeable differences in the age, body mass index (BMI), gestational age and β-hCG between the two groups (Table 1).

**Table 1:** The clinical data and hormonal detection between normal pregnancy group and threatened miscarriage groups

Item	Normal pregnancy group (*n* = 119) (Median with IQR)	Threatened miscarriage group (*n* = 96) (Median with IQR)	*P* value
Age	29 (26–32)	29 (27–32)	0.506
BMI (kg/m^2^)	21.08 (19.15–23.34)	20.28 (18.73–23.03)	0.335
Gestational age (weeks)	7.40 (6.60–11.50)	7.20 (6.40–9.83)	0.069
AEA (nM)	0.62 (0.30–1.21)	1.21 (0.72–1.83)	< 0.0001
P4 (ng/mL)	21.92 (17.48–27.83)	19.53 (13.28–24.21)	0.0013
β-hCG(mIU/mL)	30,969 (5778–100,881)	16,276 (3712–63,954)	0.125

*IQR* Interquartile range, *BMI* Body mass index, *AEA* Anandamide, *P4* Progesterone, *β-hCG* Human chorionic gonadotrophin

### Comparison of AEA, P4, β-hCG and clinical data between women with ongoing pregnancies and inevitable miscarriages

Among 96 threatened miscarriages, 58 samples were ongoing pregnancies (ongoing pregnancy group) and 38 samples were inevitable miscarriages (inevitable miscarriage group). There were no significant differences in the age, BMI, AEA and β-hCG between the two groups (Table 2). Only the median concentration with IQR of P4 was significantly lower in the ongoing pregnancy group than that in the inevitable miscarriage group, which is 15.91 (10.27-21.01) ng/mL vs. 20.59 (15.21-24.58) ng/mL.

**Table 2:** The clinical data and hormonal detection between ongoing pregnancy and inevitable miscarriage groups

Item	Threaten Miscarriages (*n* = 96)		*P* value
	ongoing pregnancy group (*n* = 58) (Median with IQR)	inevitable miscarriage group (*n* = 38) (Median with IQR)	
Age	29 (27–32)	29 (25–32)	0.237
BMI (kg/m^2^)	19.78 (18.34–22.89)	20.76 (19.50–25.14)	0.098
AEA (nM)	1.12 (0.69–1.54)	1.26 (0.86–1.97)	0.219
P4 (ng/mL)	20.59 (15.21–24.58)	15.91 (10.27–21.01)	0.0062
β-hCG (mIU/mL)	13,738 (3827–53,735)	19,941 (3586–82,214)	0.655

*IQR* Interquartile range, *BMI* Body mass index, *AEA* Anandamide, *P4* Progesterone, *β-hCG* Human chorionic gonadotrophin

### Correlation analysis among AEA, P4, β-hCG and threatened miscarriage

**Fig. 1:**
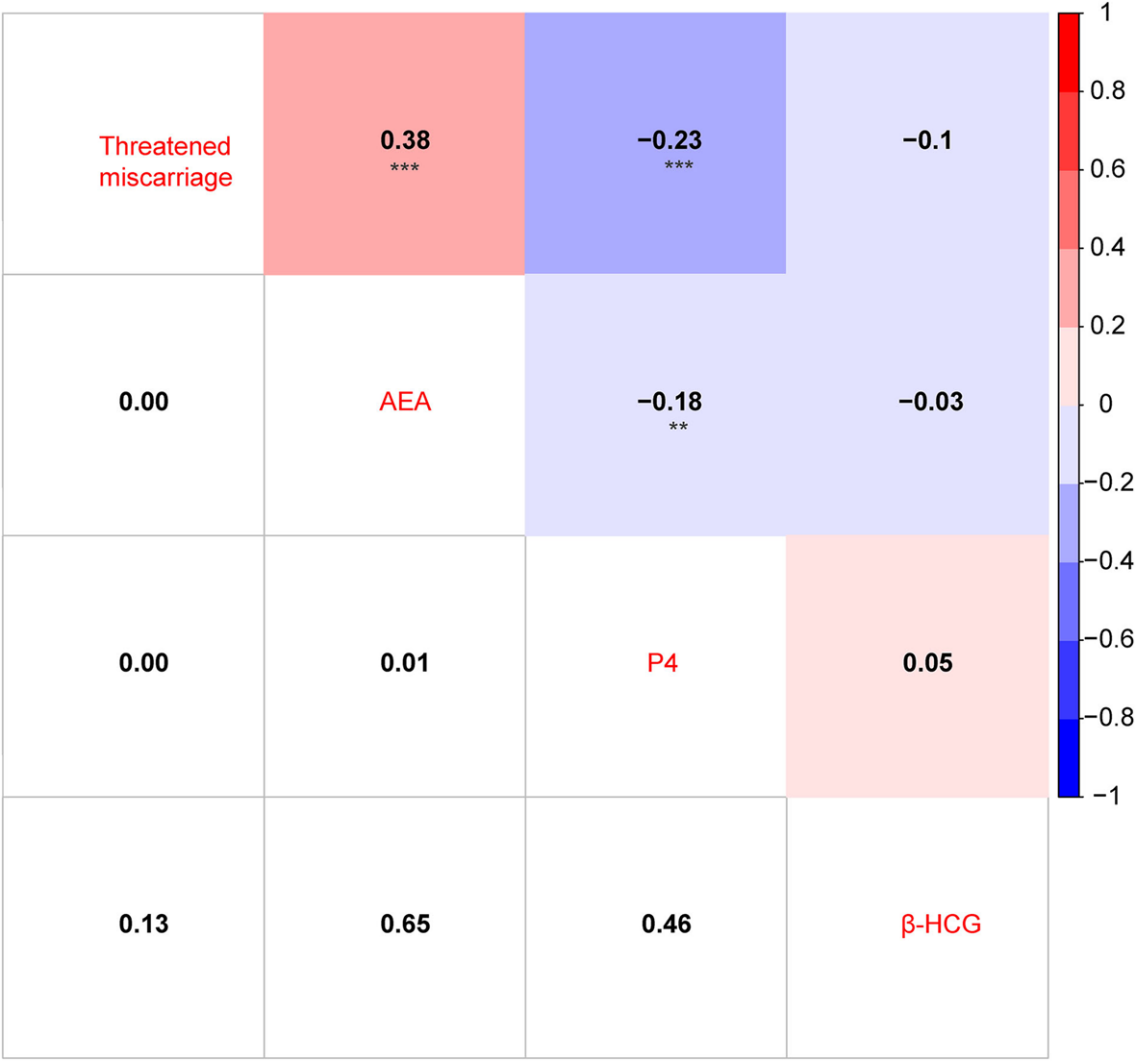
Correlations analysis among AEA, P4, β-hCG and threatened miscarriage. **p < 0.01, and ***p < 0.0001

Pearson correlation analysis was performed to evaluate the correlations among AEA, P4, β-hCG and threatened miscarriage. AEA is strongly positively correlated with threatened miscarriage (*r* = 0.38, *p* < 0.0001), while P4 is negatively correlated with threatened miscarriage (*r* = − 0.23, *p* < 0.001). Interestingly, AEA and P4 are negatively correlated with each other (*r* = − 0.18, *p* < 0.01). However, β-hCG has no significant correlation with other factors (Fig. 1). It suggests that AEA and P4 are associated with threatened miscarriage.

### Correlation analysis among AEA, P4, β-hCG and inevitable miscarriage

**Fig. 2:**
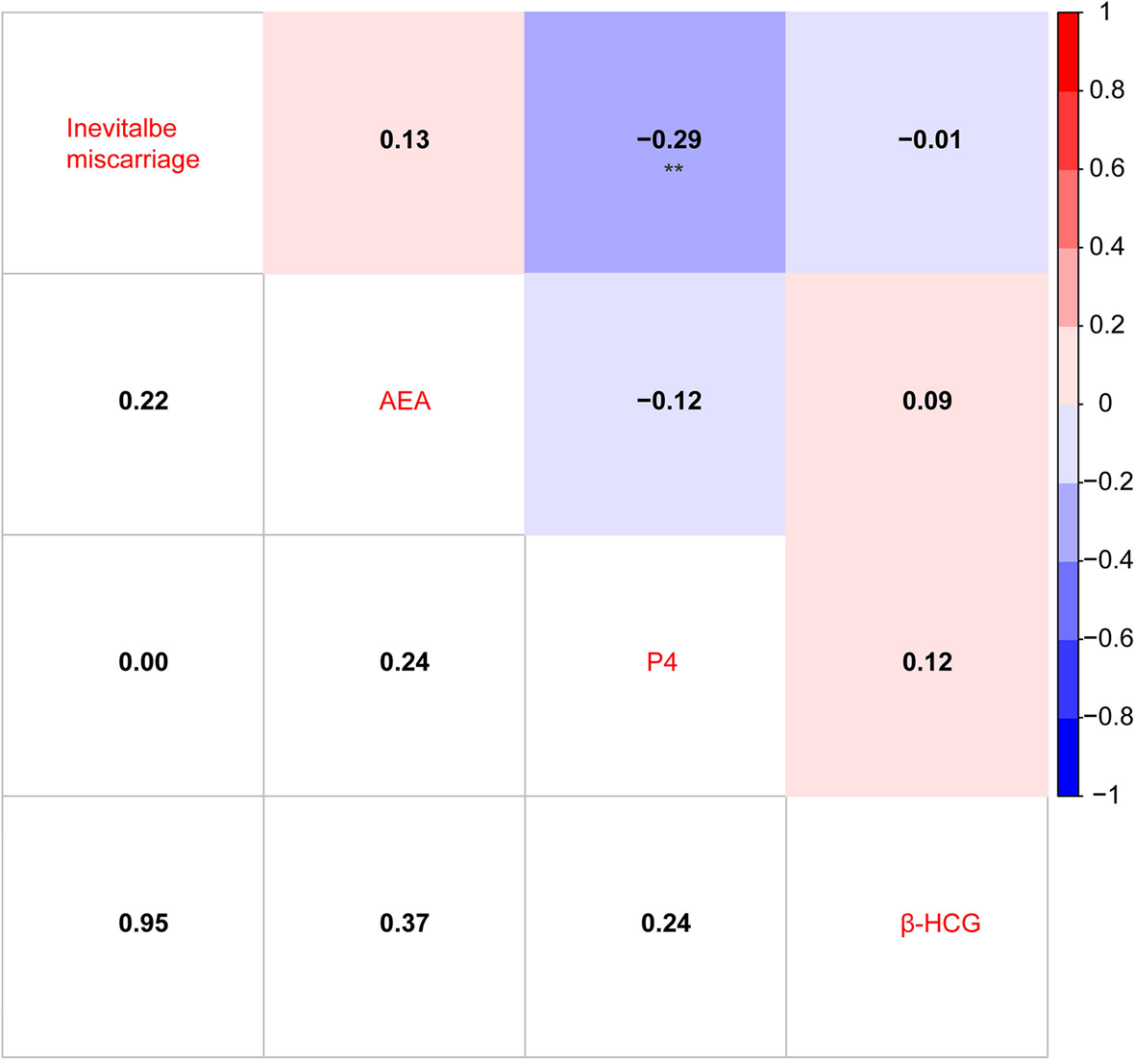
Correlations analysis among AEA, P4, β-hCG and inevitable miscarriage. **p < 0.01

Among 96 threatened miscarriage, 58 samples were ongoing pregnancies and 38 samples were inevitable miscarriages. Thus, we analyzed the correlation among AEA, P4, β-hCG tested in these patients. However, only P4 is significantly negatively correlated with the inevitable miscarriage (*r* = − 0.29, *p* < 0.01) (Fig. 2). It suggests that P4 is also associated with inevitable miscarriage.

### Comparison six predictive models

**Fig. 3:**
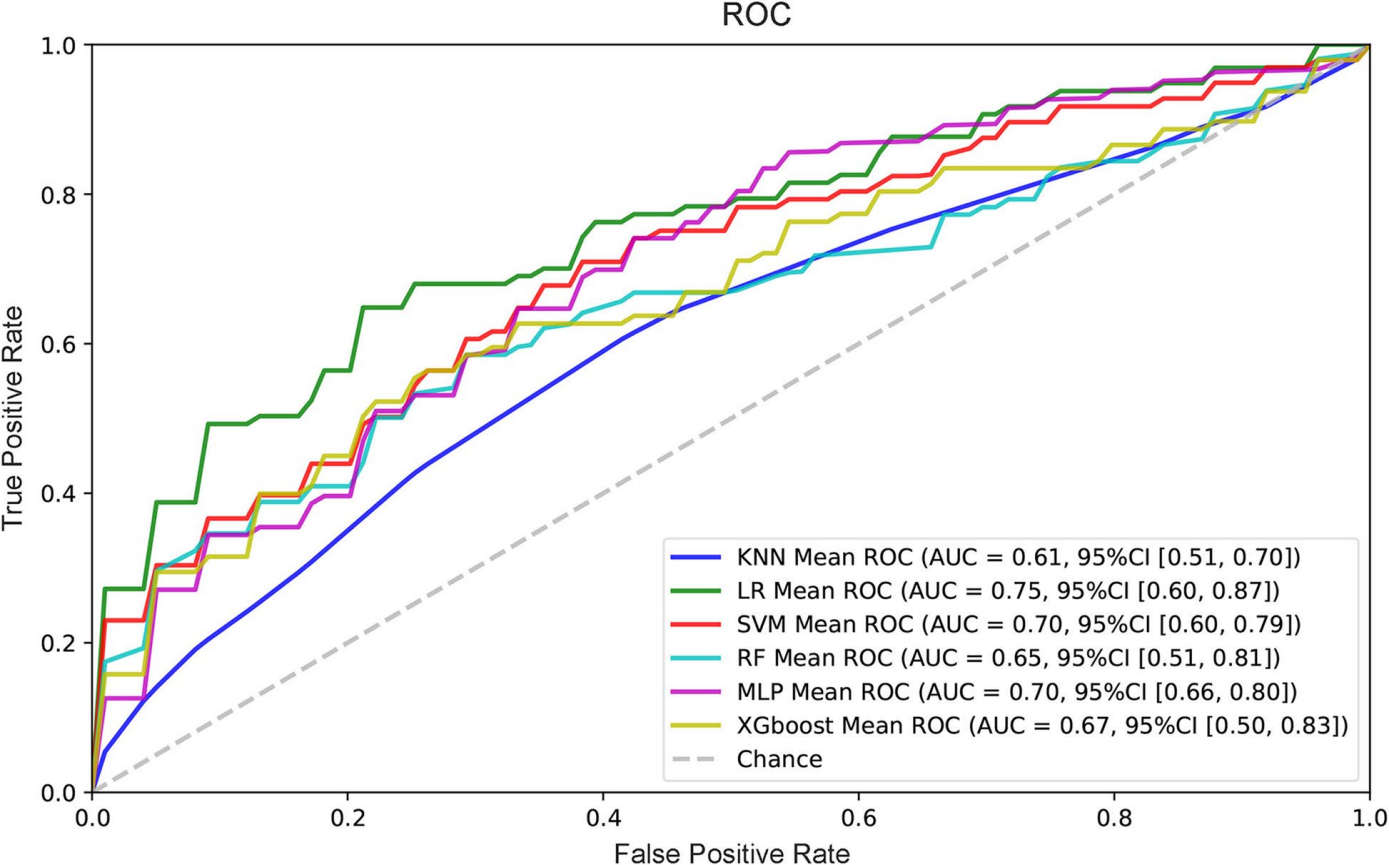
Construction 6 models to predict threatened miscarriage. The figure shows the average ROC curves of the 6 models. The mean AUC values with standard deviations of the different prediction models are shown in the box

Furthermore, we constructed six machine learning models combing AEA, P4 and β-hCG to predict the threatened miscarriage risk. Among 6 models, LR model obtained the highest AUC value 0.75 (Fig. 3), and showed the highest accuracy (0.65) and precision (0.70) (Table 3). Moreover, both SVM and MLP models had same AUC value 0.70, and the accuracy and precision were above 0.61 and 0.60, respectively. However, KNN had the lowest AUC (0.61), accuracy (0.60) and precision (0.57). The results indicated AEA, P4 and β-hCG could predict threatened miscarriage using machine learning tools.

**Table 3:** The performance of accuracy and precision in six models to predict threatened miscarriage

Models	Accuracy (95% CI)	Precision (95% CI)
KNN	0.60 (0.52–0.67)	0.57 (0.45–0.72)
LR	0.65 (0.50–0.78)	0.70 (0.48–0.98)
SVM	0.62 (0.52–0.72)	0.68 (0.48–0.89)
RF	0.64 (0.46–0.79)	0.63 (0.44-0.81)
MLP	0.62 (0.56–0.74)	0.61 (0.50–0.87)
XGboost	0.64 (0.50–0.77)	0.61 (0.47–0.79)

*KNN* k-nearest neighbors classifier, *LR* Logistic regression, *SVM* Support vector machine, *RF* Random forest, *MLP* Multilayer perceptron, *95% CI* 95% confidence interval

In order to further analyze whether combing AEA, P4 and β-hCG could predict the risk of inevitable miscarriage, 58 samples with ongoing pregnancies and 38 samples with inevitable miscarriages were enrolled in 6 predictive models. However, all models showed poor prediction ability and the AUC values were lower than 0.70. Among 6 models, LR model still obtained the highest AUC value 0.67 (Fig. S1), and the accuracy (0.61) and precision were 0.61 and 0.74, respectively (Table S1). It suggests that the prediction ability of machine learning tools combing the level of AEA, P4 and β-hCG were better in threatened miscarriage risk than that in inevitable miscarriage.

## Discussion

Threatened miscarriage is a very common problem during pregnancy and is faced with therapeutic challenges. In the present study, we used three hormones P4, AEA and β-hCG to predict threatened miscarriage in early pregnancy in order to avoid the inevitable miscarriage and help doctors to provide the active treatments for women with threatened miscarriage in the early stage.

There are various miscarriage-inducing risk factors. For example, the age of parents, female with BMI > 25 kg/m^2^, the ethnicity of black female, as well as smoking and high alcohol consumption are associated with the risk of miscarriage [1]. The association between air pollutions and miscarriage are also reported [22]. In addition, chromosomal abnormalities are found in approximately 60% of miscarried tissues [23]. In the present study, we recruited 119 healthy pregnant women and 96 samples with threatened miscarriages including 58 cases with ongoing pregnancies and 38 cases with inevitable miscarriages. However, there is no significant difference in ages and BMI between the normal pregnancy and threatened miscarriage groups, as well as between the ongoing pregnancy and inevitable miscarriage groups. Small sample size, perhaps, is one of the reasons behind this, so it is urgent to enlarge the cohort to illustrate this issue in the near future.

Besides that, the concentration of AEA was significant higher in the threatened miscarriage group than that in the normal pregnancy group, which is consistent with a previous study that AEA was higher in the non-viable pregnancy group than in the viable pregnancy group [18]. Meanwhile, β-hCG level has no significant difference between the two groups in this study, which is also similar to the previous study [18]. Furthermore, we detected the correlations among AEA, P4 and β-hCG in all participates. P4 and AEA showed a significant negative correlation with each other. A previous study has shown that the P4 enhanced the FAAH activity in lymphocytes through the transcription factor Ikaros, thus causing the AEA decreased [24]. In addition, we found that AEA was positively correlated with the threatened miscarriage, although there is no study reported yet.

With the development of artificial intelligence (AI), AI techniques like machine learning tools have been increasingly used in disease diagnosis and prediction [25, 26]. As nonlinear, fault tolerant, real-time operating AI tools, machine learning algorithms are designed to fit a set of observation by selecting the best model from a set of alternatives, and they are suitable for complex applications [27]. Ma et al. has shown that KNN, LR and XGboost models were suitable for predicting the risk of Chronic obstructive pulmonary disease [28]. Xiao et al. has established and developed LR, XGboost and Elastic Net online tools to predict chronic kidney disease progression [29].

In the present study, we used 6 machine learning tools combing AEA, P4 and β-hCG to predict the risk of threatened miscarriage. The results showed that LR, SVM and MLP models all preformed a good AUC value 0.70. According to Luo and colleagues’ research, if the model AUC is greater than 0.70, the model has high accuracy [30]. However, when applying the machine learning models constructed with AEA, P4 and β-hCG to predict the risk of inevitable miscarriage, the prediction ability are poor. Hence, machine learning combing AEA, P4 and β-hCG showed good predictive power in predicting threatened miscarriage.

There are several limitations in this study. First, the total sample size was small and unbalanced for many groups. Second, we only detect the AEA, P4 and β-hCG in the first time trimester, but not in the second and third time trimester, so we cannot compare three hormones concentrations among three stages. Third, six predictive models were used only in the training set but not in the validation set. Thus, it is urgent for us to enlarge cohorts for validation in the near future.

## Conclusion

In the present study, AEA was positively correlated with the threatened miscarriage while P4 was negatively correlated with both the threatened miscarriage and the inevitable miscarriage. Furthermore, LR model combined AEA, P4 and β-hCG showed the best performance to predict the threatened miscarriage risk. Although many studies are investigating machine learning tools with novel biomarkers as promising approaches to predict disease, in some cases, the absence of a reliable reference standard may limit the reliability of these models. In addition, establishing accurate and reliable labels for data might require more extensive follow-up. Thus, we need to validate our result in larger samples from multiple centers before the models can be applied in the clinic for predicating threatened miscarriage.

## Supplementary Information

**Additional file 1: Fig. S1.** Construction 6 models to predict inevitable miscarriage. The figure shows the average ROC curves of the 6 models. The mean AUC values with standard deviations of the different prediction models are shown in the box.

**Additional file 2: Table S1.** The performance of accuracy and precision in six models to predict inevitable miscarriage.

## Data Availability

The datasets used and/or analyzed during the current study are available from the corresponding author on reasonable request.

## Abbreviations

AEA: Endocannabinoid anandamide
P4: Progesterone
β-hCG: β-human chorionic gonadotrophin
AUC: Area under the receiver operating characteristic curve
NAPE-PLD: N-acyl phosphatidylethanolamine phospholipase D
FAAH: Fatty acid amide hydrolase
LR: Logistic regression
RF: Random forest
KNN: k-nearest neighbors classifier
MLP: Multilayer perceptron
SVM: Support vector machine
BMI: Body mass index
