# Supplementary material for: Construction of machine learning tools to predict threatened miscarriage in the first trimester based on AEA, progesterone and β-hCG in China: a multicentre, observational, case-control study
Source: BMC Pregnancy Childbirth. 2022 Sep 9;22:697. doi: 10.1186/s12884-022-05025-y (PMC9461209; doi:10.1186/s12884-022-05025-y)
Supplement: Supplementary file 1 — Additional file 1: Fig. S1. Construction 6 models to predict inevitable miscarriage. The figure shows the average ROC curves of the 6 models. The mean AUC values with standard deviations of the different prediction models are shown in the box. [file 12884_2022_5025_MOESM1_ESM.docx]

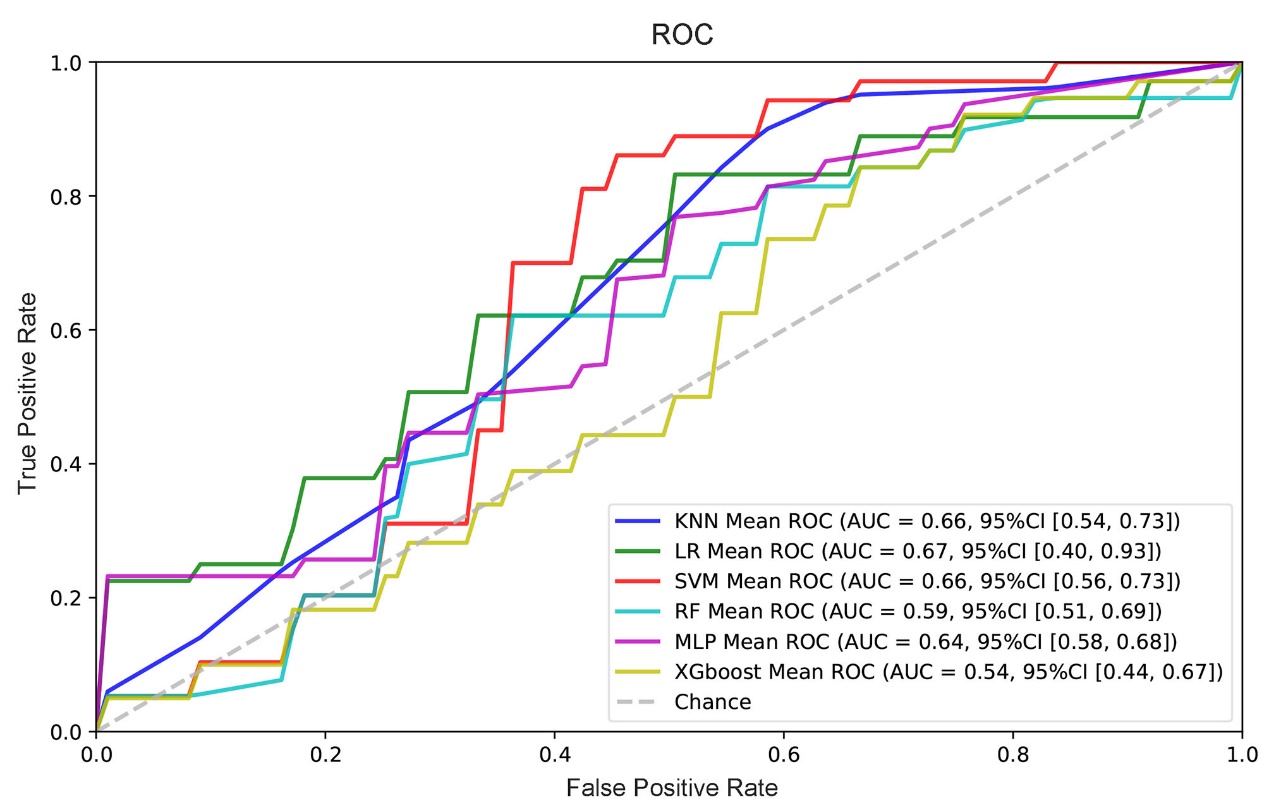
**Fig. S1** Construction 6 models to predict inevitable miscarriage. The figure shows the average ROC curves of the 6 models. The mean AUC values with standard deviations of the different prediction models are shown in the box.
