# Supplementary material for: Construction of machine learning tools to predict threatened miscarriage in the first trimester based on AEA, progesterone and β-hCG in China: a multicentre, observational, case-control study
Source: BMC Pregnancy Childbirth. 2022 Sep 9;22:697. doi: 10.1186/s12884-022-05025-y (PMC9461209; doi:10.1186/s12884-022-05025-y)
Supplement: Supplementary file 2 — Additional file 2: Table S1. The performance of accuracy and precision in six models to predict inevitable miscarriage. [file 12884_2022_5025_MOESM2_ESM.docx]

| **Table S1** The performance of accuracy and precision in six models to predict inevitable miscarriage | | |
| --- | --- | --- |
| Models | accuracy (95% CI) | precision (95% CI) |
| KNN | 0.60 (0.48-0.70) | 0.49 (0.23-0.66) |
| LR | 0.61 (0.53-0.68) | 0.74 (0.27-1) |
| SVM | 0.58 (0.48-0.72) | 0.44 (0.22-0.62) |
| RF | 0.57 (0.48-0.69) | 0.42 (0.042-0.65) |
| MLP | 0.62 (0.53-0.70) | 0.66 (0.27-1) |
| XGboost | 0.52 (0.43-0.64) | 0.40 (0.26-0.59) |

KNN, k-nearest neighbors classifier; LR, logistic regression; SVM, support vector machine; RF, random forest; MLP, multilayer perceptron; 95% CI, 95% confidence interval.
